# Intraoral appliances for in situ oral biofilm growth: a systematic review

**DOI:** 10.1080/20002297.2019.1647757

**Published:** 2019-08-06

**Authors:** Nizam Abdullah, Farah Al-Marzooq, Suharni Mohamad, Normastura Abd Rahman, Hien Chi Ngo, Lakshman Perera Samaranayake

**Affiliations:** a College of Dental Medicine, University of Sharjah, Sharjah, UAE; b School of Dental Sciences, Universiti Sains Malaysia, Health Campus, Kota Bharu, Malaysia; c Sharjah Institute for Medical Research, University of Sharjah, Sharjah, UAE; d Faculty of Dentistry, University of Hong Kong, Hong Kong

**Keywords:** Intraoral appliance, *in situ* oral biofilm, oral plaque microbiome

## Abstract

**Background**: Oral biofilms are the root cause of major oral diseases. As *in vitro* biofilms are not representative of the intraoral milieu, various devices have been manufactured over the years to develop Appliance Grown Oral Biofilm (AGOB).

**Objective**: To review various intraoral appliances used to develop AGOB for microbiological analysis, and to judge the optimal means for such analyses.

**Design**: Four databases (PubMed, Science Direct, Scopus and Medline) were searched by two independent reviewers, and articles featuring the key words ‘device’ OR ‘splint’ OR ‘appliance’; ‘Oral biofilm’ OR ‘dental plaque’; ‘*in vivo*’ OR ‘*in situ*’; ‘Microbiology’ OR ‘Bacteria’ OR ‘microbiome’; were included. The standard Reporting Items for Systematic Reviews and Meta-Analysis (PRISMA) were adopted for data gathering.

**Results**: Of the 517 articles which met the initial inclusion criteria, 24 were deemed eligible for review. The age of the AGOB, sampled at various intervals, ranged from 30 min to 28 days. The most commonly used microbiome analytical methods were fluorescence microscopy, total cell count using conventional, and molecular tools including Next Generation Sequencing (NGS) platforms.

**Conclusions**: No uniformly superior method for collecting AGOB could be discerned. NGS platforms are preferable for AGOB analyses.

The human oral microbiome comprises a veritable habitat of millions of microbes, mainly bacteria, that colonize oral surfaces including abiotic tooth surfaces and biotic surfaces such as the mucosae [1,2]. Dental plaque biofilms attached to tooth surfaces, are particularly complex mixed microbial habitats and comprise over 770 microbial species, of which 57% are identifiable, as per current data in the Human Oral Microbiome Database (HOMD). Of these at least 13% are unnamed but cultivable, and 30% are known only as uncultivable phylotypes [3].

It is also known that diseases such as dental caries, gingivitis, and chronic periodontitis result from the concerted action of multispecies biofilm communities. Although the microbial composition of dental plaques has been extensively researched for over a century, a clear picture of their composition, architecture and the metabolism remains elusive.

The nature of the complexity of the oral biofilm communities has led to the development, of a multitude of methods for their evaluation. Traditionally, conventional culture methods were employed to characterize the oral microbiota. As it is estimated that at least one-third of the latter are unculturable, there is a vast void in the understanding of natural oral microbial communities, such as plaque biofilms [3,4].

There are advantages and disadvantages of both *in vitro* and *in vivo* grown plaque methods. The former enables investigators seek the outcome of biofilm growth under standardized and simplified conditions for defined questions, and the experiments are relatively easy to conduct due to the simple standardization that may be achieved. On the contrary, the *in vivo* experiments that mimic natural oral conditions are inherently more complex but yield perhaps more realistic outcomes. Hence, many of the previous investigators have examined plaque biofilms used culture techniques that were extant during the period, using mostly *in vitro* systems, rather than *in vivo* analyses. However, the study of microbial communities within their own natural habitat is critical to improve our knowledge of disease processes such as caries and periodontitis, which are the causes for the major tooth loss in humans. Moreover, understanding plaque biofilm architecture and functionality in nature, will have profound impact on the delivery of chemicals and therapeutics for plaque biofilm control.

One of the major obstacles associated with the study of the biofilm architecture and anatomy is the difficulty to harvest intact, undisturbed natural plaque samples for analyses [5]. The literature is replete with various methods and devices for plaque biofilm collection but a few of these have addressed the quality of the biofilm obtained, and compared with the natural biofilms and artificial plaque biofilms that developed on these devices. Earlier workers have collected plaque biofilm using paper points [6], cotton rolls [7], or scalers [8–11], and clearly, these procedures are likely to disrupt the delicate three-dimensional relationships between the bacterial biomass, the extracellular matrix and the substrate [12–14], which directly influence the biofilm behavior. Hence, there is a need for a plaque biofilm collection method which does not disrupt the architecture of the biofilm [13] as a deeper understanding of the biofilm initiation, progression and maturation may open new avenues for plaque biofilm control, particularly through chemicals and antiseptics.

In order to overcome the disadvantages of the foregoing plaque collection methods, some authors have used various intraoral appliances, e.g. orthodontic appliances, as vehicles to study the naturally grown biofilm [15,16]. However, there has been, to our knowledge, no comparative analysis of the plaque biofilm collection devices, and there are but scant data on the relative superiority of one method over the other. Indeed, there is an urgent need to develop a benchmarked, universal method for evaluating *in situ* biofilm growth. Additionally, there is to our knowledge no critical review in the literature on the advantages and disadvantages of the currently available appliances, and the possible confounders that affect the outcomes. Hence, the aim of this review was to systematically review the intraoral appliances described in the literature for microbiological analysis of *in situ* oral biofilm development.

## Materials and methods

A systematic literature database search was conducted using PubMed, Science Direct, Scopus and Medline. The search included the following sets of key words:

- “Oral biofilm” or “dental plaque”
- “*in vivo*” or “*in situ*”
- “device” or “splint” or “appliance”
- “Microbiology” or “Bacteria” or “microbiome”

The search terms employed were key words classified under the general (all fields) category. The search terms were combined with an ‘OR’ and categories were combined using ‘AND’ or ‘NOT’ to create a final search query. The following filters were applied to these terms: Full text, published in the last 20 years (since 1998), English and academic journals only.

The search was conducted from March 2017 to August 2018 by two independent reviewers. Inclusion criteria were all *in vivo* or *in situ* studies on oral biofilm using intraoral device for microbiological analysis. Exclusion criteria included studies using the volunteer’s own prostheses such as denture or orthodontic appliance or *in vivo* studies on implants or fixed prostheses. Studies analyzing the microbial impact on enamel or dentine caries or intracanal bacteria were also excluded.

Focus questions were:

1. What were the materials used to construct the intraoral appliance?
2. Where was the location of the appliance? The upper or the lower jaw?
3. What were the substrates used, how many substrates were used, what were the shape and size of the substrate and the location of the substrate?
4. How many participants were involved in the study? What were their characteristics?
5. What was the age of the biofilm collected?
6. Were chemical agents used in the study? What were these agents?
7. What were the study endpoints?
8. What were the methods of analysis?
9. What were the main findings?

A database was developed to compare and assess the literatures based on the Preferred Reporting Items for Systematic Reviews and Meta-Analyses (PRISMA) statement guidelines [17].

### Search results

**Figure 1.**
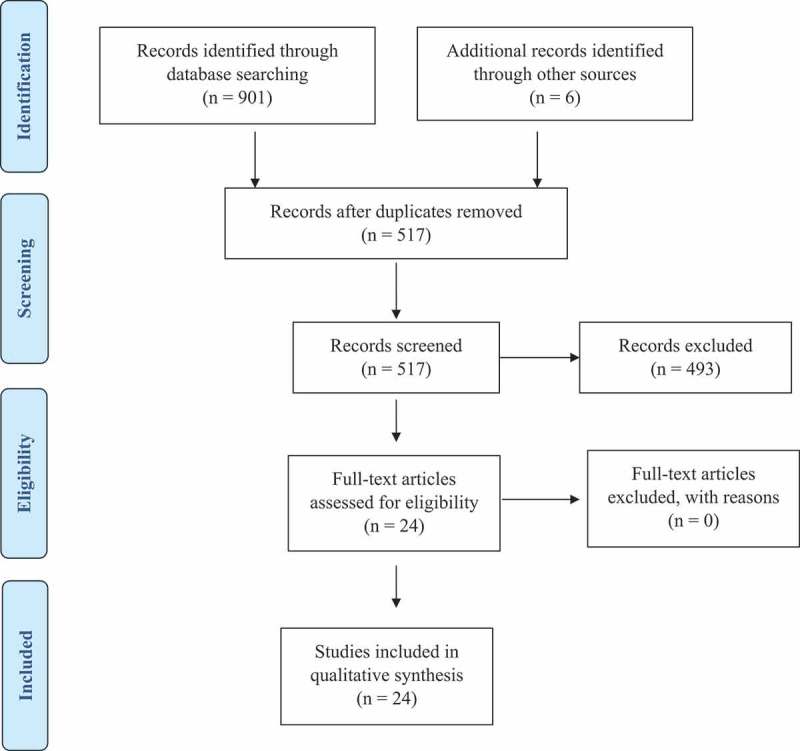
Article selection process.

Initial database search identified 901 manuscripts. Six manuscripts were identified through manual search. After removing duplicate records, 517 which met the inclusion criteria were included. After screening the abstracts, 493 articles were dismissed/eliminated based on the exclusion criteria. Finally, a total of 24 studies were included in this systematic review. The article selection process is illustrated in Figure 1.

## Analysis

A descriptive summary of the findings, tabulated and based on the focus questions are provided in Tables 1–3.

**Table 1.** Characteristics of study participants, intraoral appliances, substrates and methods used to study appliance grown oral biofilm (AGOB).

		Appliance		Substrate					Appliance grown oral biofilm (AGOB)		
Author. Year^a^	Number of subjects (age in years)	Material used	Location	Type	Location	Number	Shape	Size	Age	Outcome measures	Methods of analysis
Wood et al. 2000 [13]	8 (Not stated)	Nylon (Leeds *in situ*)	Upper jaw	Human enamel	Buccal	2	Not stated	Not stated	4 days	Architecture	CLSM^b^
Giertsen et al. 2000 [19]	11 (21–28)	Acrylic	Lower jaw	Bovine enamel	Buccal	2	Cylindrical	6.8 x 1.5mm	7 days	Total cell count Viability,	Culture, Immunofluorescence
Wood et al. 2002 [5]	4 (Not stated)	Nylon (Leeds *in situ*)	Upper jaw	Human enamel	Buccal	2	Not stated	Not stated	2,7,14 and 28 days	Architecture Thickness	CLSM
Auschil et al. 2004 [20]	8 (23–30)	Acrylic	Upper lower jaw, palatal	Glass	Buccal (upper, lower jaw) and palatal	9 -upper appliance, 6 – lower appliance	Cylindrical	3 x 2mm	48 hrs	Thickness	CLSM
Auschil et al. 2005 [21]	7 (25–29)	Acrylic	Upper jaw	Glass	Buccal	6	Cylindrical	3 x 2 mm	48 hrs	Thickness, Vitality	CLSM
Dige et al. 2007 [35]	10 (21–35)	Acrylic	Lower jaw	Glass	Buccal	Not stated	Cuboidal	4x4x1mm	6 hrs,12 hrs,24 hrs and 48 hrs	Structure, Composition	FISH, CLSM
Al-Ahmad et al. 2007 [22]	1 (27)	Acrylic	Upper jaw	Bovine enamel	Buccal	6	Cylindrical	3x2mm	1,2,3,5,7days	Thickness, Composition	FISH, CLSM
Dige et al. 2009 [14]	10 (23–36)	Acrylic	Upper jaw	Glass	Buccal	6	Cuboidal	4x4x1mm	6 hrs,12 hrs,1 and 2 days	Quantification of bacteria	FISH, CLSM
Al-Ahmad et al. 2009 [23]	6 (Not stated)	Thermoplastic	Upper jaw	Bovine enamel	Buccal	6	Cylindrical	5x1.5mm	2,6 and 12 hrs	Adherence of bacteria to device	FISH, TEM, SEM
Jung et al. 2010 [24]	6 (Not stated)	Thermoplastic	Upper jaw	Bovine dentine	Buccal	6	Cylindrical	5x1.5mm	30 mins, 2 and 6 hrs	Total bacterial count, Adhesion to substrate	Culture, FISH, CLSM, SEM, TEM
Gu et al. 2012 [25]	9 (25–42)	Acrylic	Upper jaw	Glass	Buccal	6	Cylindrical	3x1.5mm	48 hrs	Thickness, Vitality	CLSM
Tawakoli et al. 2013 [31]	6 (Not stated)	Not stated	Upper jaw	Bovine enamel	Buccal	6	Cylindrical	5x1.5mm	2 hrs	Vitality, Adherence to substrate	Culture, Florescence microscope, TEM
Langfeldt et al. 2014 [26]	32 (20–30)	Acrylic	Upper and lower jaw	Membrane filters	Buccal	8	Not stated	Not stated	1.3.5,9 and 14 days	Composition	DNA sequencing
Takeshita et al. 2015 [27]	19 (20–28)	Acrylic	Lower jaw	HA	Buccal	6	Cylindrical	5mm	1,2,3,4,5 and 7 days	Composition	Real-time PCR, DNA sequencing
Prada-López et al. 2015 [29]	5 (20–45)	Inner: EVA Copolymers Outer: Polyethylene terephthalate (IDODS)	Lower jaw	Glass	Buccal	6	Not stated	5mm	2 hrs	Thickness, Vitality, Architecture	CLSM
Quintas et al. 2015 [40]	15 (20–30)	IDODS	Lower jaw	Glass	Buccal	6	Not stated	6x1mm	2 and 4 days	Thickness, Vitality, Covering grade	CLSM
Prada-López et al 2015 [39]	20 (20–45)	IDODS	Lower jaw	Glass	Buccal	6	Cylindrical	6x1mm	2 and 4 days	Vitality, Structure, Covering grade	SEM, CLSM
Dige et al. 2016 [36]	10 (22–36)	Acrylic	Lower jaw	Glass	Buccal	8	Cylindrical	4x4x1mm	2 and 4 days	Extracellular pH	CLSM
Wake et al. 2016 [32]	10 (26–30)	Acrylic	Upper jaw	HA	Buccal	8	Cylindrical	6x1.5mm	1,4,8,12,16,24,48,60,72 and 96 hrs	Thickness, Viability, Composition	Culture, Real-time PCR, CLSM, SEM, TEM, DNA sequencing
Klug et al. 2016 [30]	25 (20–25)	Acrylic	Upper jaw	Human enamel dentine	Buccal	6	Cylindrical	6x4mm	48 hrs	Vitality, Structure, Composition	CLSM, FISH, DNA sequencing
Tawakoli et al. 2017 [38]	9 (21–41)	Acrylic	Lower jaw	Glass	Buccal	Not stated	Cuboidal	4x4x1mm	48 hrs	Spatial Distribution, Composition	CLSM, DNA sequencing
Xue et al. 2017 [33]	12 (mean 22.5 ± 2.6)	Not stated	Upper jaw	HA	Palatal	6	Cuboidal	4x4x2mm	2 weeks	Lactic acid, Vitality, Biomass	SEM, CLSM, MTT assay
Quintas et al. 2017 [37]	18 (20–45)	IDODS	Lower jaw	Glass	Buccal	6	Cylindrical	6x1mm	48 hrs	Thickness, Vitality, Covering grade	CLSM
Tomas et al. 2018 [34]	15 (20–45)	IDODS	Upper and lower jaw	Human enamel, HA, glass	Buccal	6	Cylindrical	7x2 mm	48 hrs	Thickness, Vitality, Composition	CLSM, DNA sequencing

^a^Studies arranged in chronological order.

^b^CLSM used following staining with proper live/dead fluorochromes.

**Table 2.** Characteristics of appliance grown oral biofilm (AGOB) following exposure to chemical agents.

Author. Year^a^	Chemical used	Methods	Study Endpoint	Methods of Analysis	Study Findings
Giertsen et al. 2000 [19]	3% glucose, 3% sucrose, 70 nM NaCl, 70 nM KCL, 2 mM MgCl	Double-blind cross over split mouth study design; Each subject tested 2 treatments for each test period in 2 treatment cycle.; by dipping the device 2 x daily for I min into randomly assigned test solution	Viability, CFU, Composition of specific bacteria	Culture, Immunofluorescence	NaF, zinc acetate and fluoride plus zinc acetate significantly reduced individual taxa but similar bacterial viability and total bacterial numbers were observed. However, chlorhexidine significantly reduced viability, total cell number and abundance of most of the enumerated taxa.
Auschill et al. 2005 [21]	Chlorhexidine, Amine fluoride/stannous fluoride	Observer-blind, controlled, cross-over study, rinse 2 x daily with device in the mouth with 10 ml for 1 min, morning and evening for 48 hrs	Thickness, Viability at different layers	CLSM	Both antimicrobials reduced thickness and viability significantly compared with control. However, differences between the two active solutions were not statistically different.
Gu et al. 2012 [24]	ZnCl (2.5, 5,10 and 20 mM)	Rinse twice daily for 2 mins with 10 ml of the solution with device intact for 48 hrs.	Thickness, Viability,	CLSM	Plaque index, biofilm thickness and biofilm viability treated with various conc of ZnCl reduced sig compared with control. 2.5 nM ZnCl was the lowest conc to inhibit bacteria in the outer layers, 5 mM was the lowest conc to inhibit middle layer and none could inhibit bacteria in the inner layer.
Quintas et al. 2015 [40]	Essential oil, 0.2% Chlorhexidine	Randomized, observer-masked, crossover study. Wear device for 4 days continuously. Rinse 2 x daily with device intact (20 ml for 30 sec)	Viability, Thickness, Covering grade	CLSM	Essential oil (EO) and 0.2% chlorhexidine (CHX) significantly more effective than sterile water at reducing bacterial viability, thickness and covering grade of biofilm. No sig diff bet EO and 0.2% CHX at reducing bacterial vitality. O.2% CHX showed more reduction than EO in reducing thickness and covering grade.
Dige et al. 2016 [36]	4% sucrose	Test 1: Wear device- after 30 mins. Immerse left flange in sucrose free solution (sucrose free group) for 2 mins and the right side in sucrose solution (sucrose group) for 2 mins every hour during the day for 2 days. Test 2: Same procedure repeated but exchange the immersion side	Extracellular pH of biofilm	CLSM	pH drop pattern did not differ between biofilms exposed to sucrose-free and sucrose -rich environment. Extracellular pH dropped rapidly in most sites after addition of glucose. Data suggest that pH drops in young (48 hrs) dental biofilms are independent of the sucrose supply during the growth.
Quintas et at. 2017 [37]	Essential oil with and without alcohol	Randomized, double blind crossover study. Test 1: Wear device for 48 hrs. Rinse (20 ml for 30 sec.) with device intact. Sample collection at 0, 30sec, 1, 3 5 and 7 hrs. from distal to mesial at each time point. Test 2: Rinse2 x daily (20 ml 30 sec) with device intact for 96 hrs.	Viability, Thickness	CLSM	Both antiseptics showed very high immediate antibacterial activity and substantivity *in situ* on 2-day biofilm. After 4 days both demonstrated very good antiplaque effect, but alcohol free performed better at reducing thickness and covering grade.
Xue et al. 2017 [33]	Toothpaste with and without arginine	Randomized controlled crossover study. Brushing 2 xd aily for 3 mins for 2 weeks.	Lactic acid production, MTT assay, Biomass, Vitality	SEM, CLSM	Arginine- containing toothpaste showed significant reduction of lactic acid production in both high caries and non-high caries group, but did not decrease metabolic activity, total biomass and vitality in either group.

^a^Studies arranged in chronological order.

**Table 3.** Characteristics of appliance grown oral biofilm (AGOB) not exposed to any chemical agent; Study endpoint, methods of analysis and study findings.

Author. Year^a^	Study Endpoint	Methods of Analysis	Study Findings
Wood et al. 2000 [13]	Architecture	CLSM	Plaque formed in the devices was thicker around the edges at the enamel/nylon junction (range 75–220 µm) than at the center of the device (range (35–215 µm) after 4 days.
Wood et al. 2002 [5]	Structure Density (Biomass)	CLSM	Increase in plaque density over time. CLSM images revealed that the bacterial flora in the biofilms was changing with time.
Auschill et al. 2004 [20]	Biofilm thickness	CLSM	Mean thickness was 77.6 ± 29.1 µm on the buccal site of the upper jaw 71.9 ± 26.3µm on the buccal site of the lower jaw and 52.1 ± 26.2 µm after 48 hrs. *In situ* biofilm thickness on the buccal sites was similar irrespective of the location in the oral cavity. On the palatal site the biofilm growth was significantly less.
Dige et al. 2007 [35]	Architecture, Quantification of bacteria (streptococci)	CLSM, FISH	FISH technique enabled differentiation of streptococci from other bacteria and determination of their spatio-temporal organization. Increased understanding of structure of biofilm.
Al-Ahmad et al. 2007 [22]	Biofilm thickness Composition	CLSM, FISH	Biofilm thickness increased from 14.9 ± 5.0 µm after 1 d ay to 49.3 ± 11.6 µm after 7 days. 2days- 33.6 ± 7.4 (significant), 3days- 34.3 ± 10.2 (not significant),5days-45.0 ± 6.1(significant). *Streptococcus* spp. were predominant in 1day old dental plaque and decreased significantly after 7 days. *Fusobacterium nucleatum* decreased after 2 days and increased significantly after 7 days. *Actinomyces naeslundii* significantly decreased on day 2 and 7. No significant change in *Veillonella* spp. during the study period.
Dige et al. 2009 [14]	Composition	CLSM, FISH	A notable increase in total in the total number of bacteria and streptococci over time (6,12,24 and 48hr), with a considerable interindividual variation at each time point. Streptococcal number exceeded other bacteria and over the examination period there was a relatively constant relationship between the number of streptococci and other bacteria.
Al-Ahmad et al. 2009 [23]	Composition	CLSM, FISH, TEM	The number of adherent bacteria species: *Streptococcus* species, *Veillonella* species, *Fusobacterium nucleatum* and *Actinomyces naeslundii* increased with time and all tested bacterial species were detected in the biofilm formed *in situ*. The general % composition of these bacteria did not change over investigated period but the number of streptococci containing the most frequently detected species, inc significantly with time; (2hrs: 17.7 ± 13.8%; 6hrs: 20.0 ± 16.6%; 12 hrs: 24.7 ± 16.1%).
Jung et al. 2010 [24]	Total bacterial count, Adhesion to substrate	Culture, FISH, CLSM, SEM, TEM	Initial bacterial colonization on dentine is much more pronounced than on enamel. Method employed is suitable for quantification of bacterial adhesion to dentine.
Tawakoli et al. 2013 [31]	Vitality, Adherence to substrate	Culture, TEM	The live/dead ration of CFDA/Sytox red and FDA/Sytox red was 3:2. The TEM analysis indicated that all these live/dead assays are reliable techniques for differentiation of viable and avital adherent bacteria.: BacLight, FDA/Sytox red, Calcein AM/Sytox red, and CFDA/Sytox red.
Langfeldt et al. 2014 [26]	Composition	DNA sequencing	Highly diverse entire colonization profile at 1,3,5,9 and 14 days maturation of biofilm, spread into 8 phyla divisions and in 15 different bacterial classes with a large inter-individual difference.
Takeshita et al. 2015 [27]	Composition	Real-time PCR DNA sequencing	Total no of bacteria gradually increased and reached a plateau on day 4. Microbial diversity increased between days 5 and 7.
Prada-López et al. 2015 [29]	Thickness, Vitality, Structure	CLSM	Mean vitality in the 2- and 4- day biofilms were 71% and 63%, respectively. Mean thickness were 21 µm and 28 µm respectively. There was predominance in the open and heterogenous structure whose complexity was ascending as the biofilm matured.
Prada-López, et al. 2015 [39]	Thickness, Vitality, Structure	CLSM	Thickness of biofilm after 2 and 4 days were not significantly different. The bacterial vitality changed significantly from 72.50 ± 15.50% to 57.54 ± 15.66% over time, which was in contrast to the covering grade (53.08 ± 18.03% and 70.74 ± 19.11%). The structure changed from an irregular surface and compact deepest layer with a predominance of the coccus shape to a complex structure with voids in the deepest layer and a great proportion of bacillus-shaped bacteria.
Wake et al. 2016 [32]	Thickness, Viability, Composition	Culture, Real-time PCR, CLSM, SEM, TEM, DNA sequencing	The number of viable bacteria in supragingival biofilm increased in 2 steps: Gram-positive cocci during the first 8 hrs until 16 hrs. Streptococci accounted for more than 20%. Obligate anaerobes such as *Fusobacterium, Prevotella* and *Porphyromonas* predominated after 48 hrs. Initial population of facultative anaerobic bacteria was replaced with a population of Gram-negative anaerobic bacteria during oral biofilm formation.
Klug et al. 2016 [30]	Vitality, Structure, Composition	CLSM, FISH, DNA sequencing	Compositional shifts during *in vitro* growth from zero time (T0) to 48 hrs (T3). Median values of major phyla found at T0 and T3 were 98.67 and 87.71% for *Firmicutes*, 0.01 and 3.2% for *Bacteroidetes*; 0 and 2.06% for *Proteobacteria*. 117 OTUs common to all samples. The genera *Streptococcus* and *Veillonella* (both *Firmicutes*) dominated at initial time (T0) and at 48 hrs.
Tawakoli et al. 2017 [38]	Spatial distribution, Composition	CLSM, DNA sequencing	The composition of 48 hrs biofilm sample was predominantly composed of *Streptococcus* and *Veillonella* and limited number of other genera.
Tomas et al. 2018 [34]	Thickness, Vitality, Composition	CLSM, DNA sequencing	The type of substrate, and the intraoral device/substrate position did not affect the thickness or viability of the biofilm formed on the substrate. The bacterial composition of substrate formed biofilm was similar to the tooth-formed biofilm with significant differential abundance detected in very few taxa of low abundance. The tooth brushing during the formation of substrate formed biofilm was the only factor that conditioned the thickness or bacterial viability.

^a^Studies arranged in chronological order.

CLSM: Confocal laser scanning microscope; FISH: Fluorescence *in situ* hybridization; SEM: Scanning electron microscopy; TEM: Transmission Electron Microscope; PCR: Polymerase chain reaction; EVA: ethylene-vinyl copolymers.

## Results

The number and age of the participants, the material used for fabrication of the appliance and its location in the oral cavity; the type, location, number, shape and size of substrates used in the study, and the biofilm age, outcome measures and methods of analyses are listed in Table 1. It should also be noted that most studies quoted in Table 1 based on different methodologies have reported high inter- and intra-individual differences in biofilm formation. This is not surprising as the rate of biofilm development considerably varies from individual to individual. Whereas some are `slow` plaque formers, others are `rapid` plaque formers [18]. Given the plethora of methods, substrates, subjects/cohorts used by different workers over the years (Table 1) it is extremely difficult to state whether one method is superior to another, and hence no uniformly superior method of collecting AGOB has emerged, thus far.

## The appliance and substrates used to collect biofilm

With regards to fabrication of intraoral appliance, comfort and aesthetics are important factors that should be considered for improving the participant compliance. For this reason, a number of authors have used different types of individualised acrylic splints for growing *in situ* biofilm [19–27]. However, Wood et al., [5,13] used the so-called Leeds *in situ* device, for biofilm growth, composed of a nylon ring holding an enamel substrate attached to the tooth, which was a modification of a similar previously described appliance by Robinson et al. [28]. These devices were bonded to free buccal surfaces of the first or second upper molars by means of a composite resin, providing a stagnation site for the formation of the biofilm. More recently Prada-Lopez et al., [29] developed the Intraoral Device of Overlaid Disk-holding Splint (IDOD) in the lower jaw of the volunteers. The device consists of a soft flexible inner splint and a more rigid outer splint carrying the glass substrate in between the two splints. Most workers placed the appliance in one jaw only, either the upper jaw [5,13,14,21–25,30–33] or the lower jaw [19,27,29,30–34,37]. However, Auschill et al. [20], Langfeldt et al. [26] and Tomas et al. [34] placed the appliance in both jaws to investigate the differential characteristics of the biofilm growing in both the upper and lower jaws.

Different solid substrates with varying properties have also been used in these studies for instance human enamel in the Leeds *in situ* device [5,13], human enamel-dentine slab [30], bovine enamel/dentine [19,22–24,31], polished glass [14,20,21,25,29,34–40], hydroxyapatite discs [27,32–34], or membrane filters [26]. Although the roughness of the surface of the substrate and its free energy are considered important factors for *in vivo* growth of the biofilm, Auschill et al. [20] and Netuschill et al. [41] found no major differences in the thickness of 48-h biofilm grown on enamel or glass discs. Additionally, some authors opined using glass preferentially, to obviate optical disturbance, associated with autofluorescence of enamel [41].

The number of substrates used in each experiment also varies substantially from 2 [5,13,19] to 15 [20]. Most workers used six substrates on the buccal side of the appliance, placing three substrates on each side of the jaw [14,21–25,27,29–31,33,34,37,39]. The latter workers employed either cylindrical or cuboidal form substrates. The size of the substrate was variable; as for the cylindrical discs the diameter varied from 3 mm [20–22,25] to 7 mm [34], while the height varied from 1 mm [14,35,36,38,40] to 4 mm [30]. Interestingly, Wood et al. [13] observed significant variations in the thickness of the biofilms generated over the 4-day period between each disc, depending on the substrate architecture. For instance, the biofilm was thicker at the enamel disc/ring junction (depth 75 to 220 µm) and thinner towards the center (depth 35 to 215 µm). This could be rather based on the mechanical protection of the ring system around the sample and should be taken into account in the interpretation of such systems.

All authors quoted in this review placed the substrates on the buccal aspect of the jaw when they used upper or lower appliance except for Xue et al. [33], who placed the hydroxyapatite substrate on the palatal aspect covered with plastic mesh to protect the device from mechanical disturbances while allowing free contact with saliva. Auschill et al. [20] however, demonstrated that the mean thickness of 48-hr biofilm ranged from 14 to 150 µm and was not affected by the location of the removable appliance in the oral cavity (maxillary buccal region versus mandibular buccal region) or by the position of the substrate (distal versus mesial; right versus left). In addition, Tomas et al. [34] reported the position of the intraoral device and substrate did not affect the thickness and vitality of the biofilm formed on the substrate.

These rather conflicting findings on the thickness of biofilms, surface colonization and bacterial adhesion in the *in situ* devices described above could be due to the many variables involved including intraoral locale and variations in salivary flow and dietary habits of individuals. Hence future workers must pay heed to these confounders when conducting *in situ* biofilm experiments.

## Participants and biofilm age

Participants in the 24 studies analyzed healthy volunteers with an age range of 20–45 years. Study volunteers included dental students or institutional staff from either the medical or the dental schools. It is clear that these volunteers were chosen in view of accessibility, and ease of intermittent biofilm collection and processing either during or immediately after the experiment. The number of participants recruited ranged from a single volunteer [22] to 32 [26]. However, several studies have reported a marked inter-individual variability in the characteristics of *in situ* biofilms [13,20,35], as mentioned above.

The inclusion and exclusion criteria employed were similar in most studies. Inclusion criteria included participants who were systemically healthy with good oral health, minimum of 24 permanent teeth present, no evidence of gingivitis or periodontitis and absence of untreated caries. Exclusion criteria applied were smokers or ex-smokers, presence of dental prostheses or orthodontic appliance, antibiotic treatment or routine use of oral antiseptics in the past 3 or 6 months. Tawakoli et al. [38] excluded pregnant and breastfeeding women in their study; to avoid hormonal interference with the microbial ecology. Some studies, however, did not mention any exclusion criteria [5,13,35]. Several studies included both male and female volunteers [14,19,25,33,35,36,38]. Klug et al. [30] for example, used only male volunteers, while other studies did not mention the gender of the participants [5,13,20,21,23,26,27,29,31,37,40].

The duration of biofilm growth examined varied from 30 min [24] to 28 days [5], depending on the type of biofilm analyzed. Several studies collected biofilm at a single point of time; either after 2 h [29,31], 2 days [25,30,32–34,37], 4 days [13] or 7 days [19] to identify the characteristics of the biofilm only at one-time point. In other studies biofilm was grown for a prolonged duration with periodic biofilm collection [5,14,21–23,25,26,28,29,38,40].

In terms of biofilm thickness, Al-Ahmad et al. [22] demonstrated that the mean biofilm thickness after 1 day was 19.9 ± 5.0 µm and increased significantly after 2 days (33.6 ± 7.4 µm) while after 3 days the increase was insignificant. Not surprisingly, the degree of microbial coverage, as well as the composition of the biofilm microbiota varied considerably between different individuals at different time intervals [14,35].

## Characteristics of oral biofilm following exposure to chemical agents

In translational terms, the model systems once standardized and calibrated should be ideal for evaluating the effect of chemical agents on the biofilm microbiota. Several workers have studied the ecological changes of biofilms exposed to various antimicrobial agents (Table 2). The main outcome measures evaluated in the latter studies were bacterial viability (live/dead ratio) and bacterial biomass or thickness of the biofilm, analyzed using confocal laser scanning microscope.

The main chemical agents used in these studies were chlorhexidine gluconate, amine fluoride/stannous fluoride, zinc chloride, alcohol and essential oil [21,25,37,40]. Both chlorhexidine and amine fluoride/stannous fluoride significantly reduced the biofilm thickness and biofilm viability compared to controls, but the differences between the two agents were not significant [21]. In another study, Gu et al. [25] evaluated zinc chloride, at 2.5, 5, 10 and 20 mM concentrations, and noted significant reduction in the plaque index, biofilm thickness and biofilm viability compared with controls. They also evaluated the effect of zinc chloride on various biofilm layers, and reported that 2.5 mM was the lowest concentration to inhibit the outer layer, 5 mM was the lowest to inhibit the middle layer while none of the used zinc chloride concentrations could inhibit the bacteria in the inner layer [25].

Antiplaque formulae agents, based on essential oils either with or without alcohol, showed very high immediate antibacterial activity and substantivity in a 2-day biofilm. After 4 days both demonstrated very good antiplaque effect, but alcohol-free essential oil was better at reducing the biofilm thickness [37].

Apart from the above, some have evaluated the effect of dietary sucrose on the artificial biofilm grown *in situ*. In a recent study, Dige et al. [36] compared the profiles of pH drops in plaque biofilms exposed to 4% sucrose. They removed the device with the grown biofilm from the oral cavity and immersed it in a sucrose solution. The authors noted no difference in pH between the test and the sucrose-free sample, which was a rather surprising finding.

Xue et al. [33] evaluated the lactic acid production in an *in situ* biofilm after exposure to toothpaste, with and without arginine. They demonstrated a significant reduction in lactic acid production, but not a decrease in metabolic activity, total biomass or vitality, when using toothpaste containing arginine in two cohorts with high and low caries activity. In these studies, the chemical agents were exposed to the biofilm directly either *in vitro* by dipping the device into the test solution for 1 min twice a day [19], for 2 min during the day for 2 days [36], or while the device was *in situ* in the oral cavity, by brushing [33] or rinsing with the solution [25,37,42]. In the latter instance, oral rinsing was performed twice daily in the morning and in the afternoon, either for 1 or 2 min.

## Techniques used in *in situ* microbiome analyses: microbiological techniques

Various microbiological techniques have been employed over the years to analyze the growth of *in situ* plaque biofilms, and the salient data are tabulated in Tables 2 and 3.

The oldest, the standard, and the most widely used method to analyze biofilm growth is to determine the biofilm cell viability and quantifying the bacterial growth in terms of colony-forming units (CFUs) on culture plates using either differential or universal media. For instance, Jung et al. [24] and Giertsen et al. [19] used selective culture media and CFU counts with the help of a stereomicroscope to evaluate biofilm growth.

Wake et al. [32] determined that the viable cell counts of biofilm cultures under aerobic conditions increased rapidly during the first 12 h and increased gradually thereafter. After a significant cell growth increment between 48 and 72 h, the population of viable cells plateaued. However, such culture-based methods have several drawbacks as it is estimated that a third of oral microbiota is unculturable [3,4,18]. Moreover, it is unclear which proportion of the biofilm microbiota is viable. With the advances in microbe identification and visualization and imaging systems recent workers have utilized the following advanced techniques for studying AGOB [43]:

- **Biofilm growth and viability testing with live/dead stain and Confocal Laser Scanning Microscopy**

Confocal Laser Scanning Microscopy (CLSM) has been widely used over the last two decades or so to evaluate AGOB. CLSM provides a hitherto unknown means of studying biofilm viability and structural features, and in particular, the spatial orientation of the various layers of the biofilm. Additionally, as membrane integrity is a surrogate marker of bacterial cell viability, this feature is utilized to differentiate viable from nonviable cells in AGOB. The method, called live/dead stain, uses red-fluorescent, membrane-impermeable, nucleic acid stain – propidium iodide (PI), and hence can penetrate only dead cells with damaged cytoplasmic membranes, whereas the counterstain, green-fluorescing SYTO 9 can penetrate viable cells with intact, but also non-viable bacteria with damaged cell membranes. Thus, viable cells are stained by SYTO 9 which fluoresces green, while the nonviable cells are stained with propidium iodide which fluoresces red [21,25,29,33,37].

CLSM has also been widely used to observe biofilms in three dimensions (3D) in either static or dynamic growth milieus. These high-quality, time lapse images of biofilm can then be used for systematic collection of data for digital image analysis, and subsequent evaluation of biofilm growth and physiology [5,13,29,30,35,40].

b. **FISH and CLASI-FISH**

While genome-sequencing methods aim to catalog the resident microbe constituents of AGOB, they cannot reveal the bacterial community architecture, and the spatial arrangement of the constituents. It has been shown that the use of a combination of different microbiological visualization techniques is the only means to achieve a realistic representative of spatial distribution of *in situ* biofilms. In order to examine such spatial organization of the microbial constituents in a biofilm, and their relationships with the neighboring microbiota within this ecosystem, a technique known as fluorescence *in situ* hybridization (FISH) has been employed in several studies [22,24]. Many have analyzed such relationships and also fluxes in specific members of microbial populations over time [14,22–24,30,35]. Dige et al. in two of their studies [14,35], applied 16S rRNA-targeted oligonucleotide probes to identify streptococci and other bacteria, while Al-Ahmad et al. [22] used multiplex FISH to identify simultaneously the dynamics of four important bacterial constituents in the oral biofilm. They concluded that FISH was an appropriate method for quantifying initial biofilm formation *in situ*, and the proportion of streptococci increases during the first 12 h of bacterial adherence [22]. Dige et al. [35] collected the biofilm after 6, 12, 24 and 48 h to study initial formation of biofilm by applying 16S rRNA-targeted oligonucleotide probes for identification of bacteria. Between 24 and 48 h, the predominant colonizers were streptococci. During the 6 to 12-h period the biofilm growth manifested as small chains of streptococci, which on further incubation developed into simple, monolayers. However, other non-streptococcal species including *Actinomyces naeslundii, Veillonella* spp. and *Fusobacterium nucleatum* were also detected at the early stages of biofilm formation in some studies. Klug et al. [30] employed FISH technique to obtain detailed information on cell viability and to confirm the biofilm composition evaluated by pyrosequencing techniques.

FISH technology, however, has limitations as only three to four types of organisms can be identified. As this is a woeful inadequadequacy for the study of plaque biofilm with a multitude microbiota, a relatively new technique called CLASI–FISH has been developed [44]. CLASI–FISH technique combines combinatorial labeling and spectral imaging (CLASI) with fluorescence *in situ* hybridization (FISH). In this technique, each bacterial genus can be labeled with two fluorophores, which allow many color combinations [45,46]. Hence, CLASI-FISH technology awaits exploitation by future workers evaluating the ultrastructure of AGOB.

c. **Scanning Electron Microscopy (SEM) and Transmission Electron Microscope (TEM)**

Several workers have employed SEM/TEM to analyze adherence and growth of AGOB [24,32,33]. Wake et al. [32] observed the presence of coccal forms after 8 h, and filamentous bacteria after 12 h while Xue et al. [33] recently analyzed the structures of AGOB in high caries and non-caries groups using SEM and found that they were similar. Tawakoli et al. [31] confirmed the ability of fluorescence-based live/dead staining in detecting viable/non-viable cells using fluorescence microscopic visualization, as well as TEM. They concluded that the tested live/dead stains can be used for evaluation of the early phase of AGOB and adoption of other methods, such as TEM, complements well the fluorescence imaging.

d. **Polymerase Chain Reaction (PCR)**

Only two studies in this review used quantitative real-time PCR (qPCR) technology to analyze AGOB at different time points. Wake et al. [32] used real-time PCR to determine the quantity of bacteria present at 0, 12, 24, 36, 48, 60, 72, 84 and 96 h. They found that the quantity of bacteria increased significantly from 1 hour up to 72 h and then plateaued thereafter. They also reported a divergence between the viable bacterial cell counts (under aerobic or anaerobic conditions) with the total number of bacteria detected by real-time PCR in the AGOB confirming the observations, reported above [31]. Takeshita et al. [27] also used real-time PCR to determine total bacteria in AGOB and noted quantitative increase in bacterial biomass of AGOB over time, reaching a plateau population on day 4.

e. **Next-Generation Sequencing**

Recent advances in molecular microbiological techniques have paved the way to analyze the human oral microbiome in great detail, and Next-Generation Sequencing (NGS) platforms have played a major role in this context through identification of various hitherto unknown phylotypes of unculturable bacteria. Many NGS platforms have been successfully used for the 16S rRNA-based metagenomic analysis of the oral microbiome. Older NGS methods, like pyrosequencing (Roche, 454) is based on the detection of pyrophosphate released during DNA synthesis [47]. Newer NGS methods are based on the detection of fluorescently labeled nucleotides during sequencing by synthesis in Illumina platforms (such as MiSeq and HiSeq) [47] or the detection of pH change onto a semiconductor chip to identify the sequenced nucleotides in an Ion Torrent platform [48]. These methods were used to perform taxonomic profiling by 16S rRNA amplicon sequencing of different hypervariable regions of bacterial genomes to identify different phylotypes [49,50].

Six studies quoted in this review analysed the composition of AGOB utilising NGS methods [26,27,30,32,34,38]. The basic findings from these studies were similar to data derived from conventional culture methods, as all observed increasing and complex diversity of the bacterial population over time. Wake et al. [32], for instance, using an NGS Illumina platform demonstrated that the genera *Streptococcus* and *Neisseria* were predominant in the early phase of biofilm formation on hydroxyapatite disks, with the emergence of Gram-negative anaerobic bacteria such as *Fusobacterium, Prevotella* and *Porphyromonas*, after 48 h. The phylum *Firmicutes* was the pioneer biofilm colonizers (primary colonizers) and *Fusobacteria* and *Bacteroidetes* increased thereafter up to 48 h [30,32]. A similar observation was made by Takeshita et al. [27] who reported that the deposition of the bacteria on a hydroxyapatite disk was time-dependent. At early stages of the biofilm (until day 4), facultative anaerobic bacteria such as *Streptococcus, Neisseria, Abiotrophia, Gemella*, and *Rothia* were dominant, whereas obligate anaerobes, such as *Porphyromonas, Fusobacterium*, and *Prevotella*, and facultative anaerobic *Capnocytophaga* were dominant after 4 days of biofilm maturation.

Both Klug et al. [30] and Tawakoli et al. [38] found that the composition of 48-h biofilm sample was predominantly composed of *Streptococcus* and *Veillonella* and a limited number of other genera, while Tomas et al. [34] demonstrated that the most abundant genera in biofilm samples were *Streptococcus, Fusobacterium, Veillonella, Neisseria, Gemella, Prevotella, Alloprevotella, Porphyromonas, Aggregatibacter*, and *Leptotrichia.*

It is of practical and clinical interest to note that biofilms developing on an artificial substrate such as hydroxyapatite differ from those on enamel surfaces. For instance, *Streptococcus* and *Fusobacterium* were the most abundant genera on the artificial substrate hydroxyapatite (56.95%-23.62% and 65.92%-13.06%, respectively), while on enamel surfaces *Streptococcus* (45.69%-19.72%), *Fusobacterium* (56.91–6.81%), *Veillonella* (27.72–2.38%) and *Neisseria* (12.12%-3.37%) were the most abundant [34]. A puzzling feature of these NGS studies is the very high variations in operational taxonomic units (OTUs) in the oral biofilms over time between individuals. For instance, Langfeldt et al. [26] noted that OTUs ranged from 7 to 130 per sample. This could be a real difference in biofilm sample or apparent variation due to poor standardization of the NGS technology. It is well known that the output of NGS studies is highly technique sensitive depending on the quality of the primers used as well as the DNA purity [48,51].

## Discussion

Dental plaque biofilms are the prime movers of the most common oral pathologies such as dental caries and periodontal disease [18,52,53]. It is therefore, critically important to have a firm understanding of biofilm biology, and the first step in this direction is to study the colonization profiles and the architecture of this complex community of organisms in its natural habitat *in situ*. Hence, modeling the *in situ* oral biofilm development still remains a cornerstone, though a yet elusive goal, in oral microbiome research. Such models have played an important role, particularly in cardiology, from testing the effects of new caries prevention methods, to developing new caries-preventing products.

As seen in this review the design of the oral biofilm models varies from simple to sophisticated according to the purpose of the investigation. Over the years, a number of studies have yielded varied results using these complex microbial culture models that yield biofilms which closely mimic natural dental plaque [54,55]. As mentioned, there are pros and cons of both the *in vitro* and *in vivo* grown plaque biofilms. *In vivo* biofilm is grown under natural oral conditions that are inherently more complex but yield more realistic data; thus, it gives better representation of the normal oral microbiota composed of hundreds of species. On the other hand, *in vitro* biofilm can be grown under standardized and simplified conditions for defined questions, and the experiments are relatively easy to conduct as less microbial species are involved. Early workers preferred the conventional microbial culture methods in the laboratory because they provide a controllable and a reliable environment, and obviating the necessity to obtain ethical clearance, for human studies. However, such models are a compromise between the reality of the oral cavity and the simplicity of the *in vitro* environment [56]. It is clear that cultivation of bacterial biofilms in an artificial environment *in vitro* is unlikely to reflect the physiological conditions extant in the oral cavity, and may not reproduce the architectural, physiological and constitutional features of the *in vivo* biofilms.

The appliances designed to study *in situ* biofilms must allow free flow and contact between saliva and the substrate, to permit natural plaque biofilm development, and at the same time protect it from mechanical disturbances. However, some appliances appear to fail due to inherent disturbances associated with the muscular action of the tongue and cheek, thus yielding inconsistent results. Comfort and aesthetics are also very important factors that need to be considered for effective compliance by the volunteers wearing the appliance.

As for the location of the appliances reviewed here, 12 were worn in the upper jaw [5,13,14,21–25,30–33], while nine appliances were placed in the lower jaw [19,27,29,35–40], and another three were placed both in the upper and lower jaw [20,26,34]. According to some authors, the site of appliance placement either in the upper or lower jaw, may not be critical. Auschill et al. [20] demonstrated similar biofilm thickness at different locations in the buccal region of the upper and lower jaws. However, from the viewpoint of comfort, subjects may prefer wearing the appliance in the upper jaw since the lower jaw is highly mobile and the tongue movements are likely to dislodge the appliance.

The nature of substrates used to collect the biofilm would certainly influence biofilm development. A variety of substrates have been employed in studies ranging from biological based substrates that include human or bovine enamel and dentine, to non-biological synthetic substrates such as glass, hydroxyapatite and polystyrene. Clearly, acrylic (polymethyl methacrylate) appears to be the popular material of choice used in construction of these devices, as 12 out of 24 studies described in the current review [14,20–22,27,30,32,35,36] used the latter substrate.

Enamel, when used as a substrate, could be either of human or bovine origin, and is preferably employed for evaluating cariogenic biofilm development [57]. However, in research related to endodontic therapy, both human [58] and xenogenic [59] dentine were popular substrates. Nevertheless, many workers have used synthetic substrates such as polymethyl methacrylate, glass and hydroxyapatite as substrates as these provide standardized and uniform surface features compared with enamel and dentine. Also, it is well known that oral bacteria adhere well to glass surfaces and develop profuse biofilms [60]. Many workers have also used hydroxyapatite in the form of either beads [61,62] or discs [63,64] for AGOB development. Hydroxyapatite represents the chemical and structural architecture mimicking dental tissues, thereby avoiding the need to use human enamel/dentine. Yet, other workers have used polystyrene as their substrate surface for studying biofilm formation. Loo et al. used polystyrene substrate to study *Streptococcus gordonii* biofilm and particularly to identify the genes that code for biofilm phenotypes [65]. Others applied 96-well polystyrene plates to investigate the effects of antibiotics on biofilm formation [66].

Synthetic substrates such as glass, polystyrene and hydroxyapatite have additional advantages as they can be shaped according to the required design and are easy to sterilize. Furthermore, biological substrate such as bovine and human enamel and dentine may be more difficult to standardize in terms of their size, shape and profile, and although they could be sterilized, may still carry the risk of transmitting bovine zoonotic infection or other viral-borne threats [39,67].

The design and shape of the substrates used in the evaluated studies vary from flat discs, cylindrical or cuboidal shapes, but these configurations may not be significant as long as their surface texture is standardized. However, their sizes will certainly influence the amount of plaque collected since a larger sized/diameter substrate will permit a bigger volume of biofilm growth. Irrespective of the design, the substrates could be attached to the appliance by various means such as sticky or red wax [20–22,27,35], by using impression material [23,24,31], or by sticking to the gingiva with surgical sutures [26].

## Conclusion

The most critical aspect of AGOB in clinical terms, is to identify the growth of biofilm and its constituents, and eventually to evaluate the effect of various anti-biofilm agents. Advances in CLSM and CLASI-FISH technology now permit three-dimensionally reconstructed images of the biofilm, allowing visualization of its depth and width [30,34,37,40], while NGS studies of AGOB provide new clues to the unculturable organisms that lurk in these consortia. It is hoped that new biofilm management therapies can be evaluated using the AGOB aided with the new combinatorial technologies. For instance, visualization of the oral biofilm with CLSM or CLASI-FISH coupled with quantitative and qualitative assessment via newer third-generation sequencing may help elucidate the true nature of these complex microbial consortia that have eluded our grasp thus far.

Finally, given the plethora of methods, substrates, and subjects/cohorts used by different workers reviewed here it is extremely difficult to state whether one method is superior to another, and hence no uniformly superior method of collecting AGOB has emerged. Nevertheless, our review of the methodology, should assist the novice in selecting the best method for his/her own experimental needs for AGOB collection. However, in terms of the analysis of the AGOB microbiome/microbiota, it can be safely concluded that NGS and the rapidly emerging, high fidelity, so-called `third-generation` sequencing techniques will be the future.
